# Macrophages and scavenger receptors in obesity‐associated non‐alcoholic liver fatty disease (NAFLD)

**DOI:** 10.1111/sji.12971

**Published:** 2020-09-21

**Authors:** Sine Kragh Petersen, Orsolya Bilkei‐Gorzo, Olivier Govaere, Anetta Härtlova

**Affiliations:** ^1^ Department of Microbiology and Immunology at Institute of Biomedicine Wallenberg Centre for Molecular and Translational Medicine University of Gothenburg Gothenburg Sweden; ^2^ Translational and Clinical Research Institute Faculty of Medical Sciences Newcastle University Newcastle upon Tyne UK

**Keywords:** lipid metabolism, macrophages, NAFLD

## Abstract

With an increase in sedentary lifestyle and dietary over nutrition, obesity has become one of the major public health problems worldwide and is a prevalent predisposing risk factor to non‐alcoholic fatty liver disease (NAFLD), the most common chronic liver disease in Western developed countries. NAFLD represents a series of diseased states ranging from non‐alcoholic fatty liver (NAFL) to steatohepatitis (NASH), which can lead to fibrosis and eventually to cirrhosis and hepatocellular carcinoma. Currently, the only effective treatment to cure end‐stage liver disease is liver transplantation. Macrophages have been reported to play a crucial role in the progression of NAFLD, thereby are a potential target for therapy. In this review, we discuss the current knowledge on the role of macrophages and inflammatory signalling pathways associated with obesity and chronic liver inflammation, and their contribution to NAFLD development and progression.

## 1 INTRODUCTION

Over the last decade, NAFLD—recently also defined as Metabolic Associated Fatty Liver Disease, MAFLD—has become the predominant cause of chronic liver disease worldwide.^1^ In fact, the majority might be due to rapid increase of overweight and obesity. In 2016, the World Health Organization (WHO) estimated that close to 2 billion adults worldwide were obese or overweight and this number is continuing to rise as up to 38 million children under the age of 5 are overweight.^2^ NAFLD constitutes a spectrum of liver diseases ranging from simple fat accumulation in the liver (NAFL, isolated steatosis) to inflammatory state, non‐alcoholic steatohepatitis (NASH), which can lead to fibrosis (scaring of tissue) and ultimately to cirrhosis (permanent tissue damage) and hepatocellular carcinoma (HCC).^3^ The prevalence of NAFLD is estimated about 20%‐40% worldwide, and it is anticipated to increase exponentially due to high prevalence of obesity.^1^ Moreover, the presence and severity of NAFLD are associated with the risks of serious extrahepatic diseases, such as hypertension, insulin resistance, dyslipidemia, colon cancer, chronic kidney diseases and type II diabetes.^4^, ^5^

**Figure 1:**
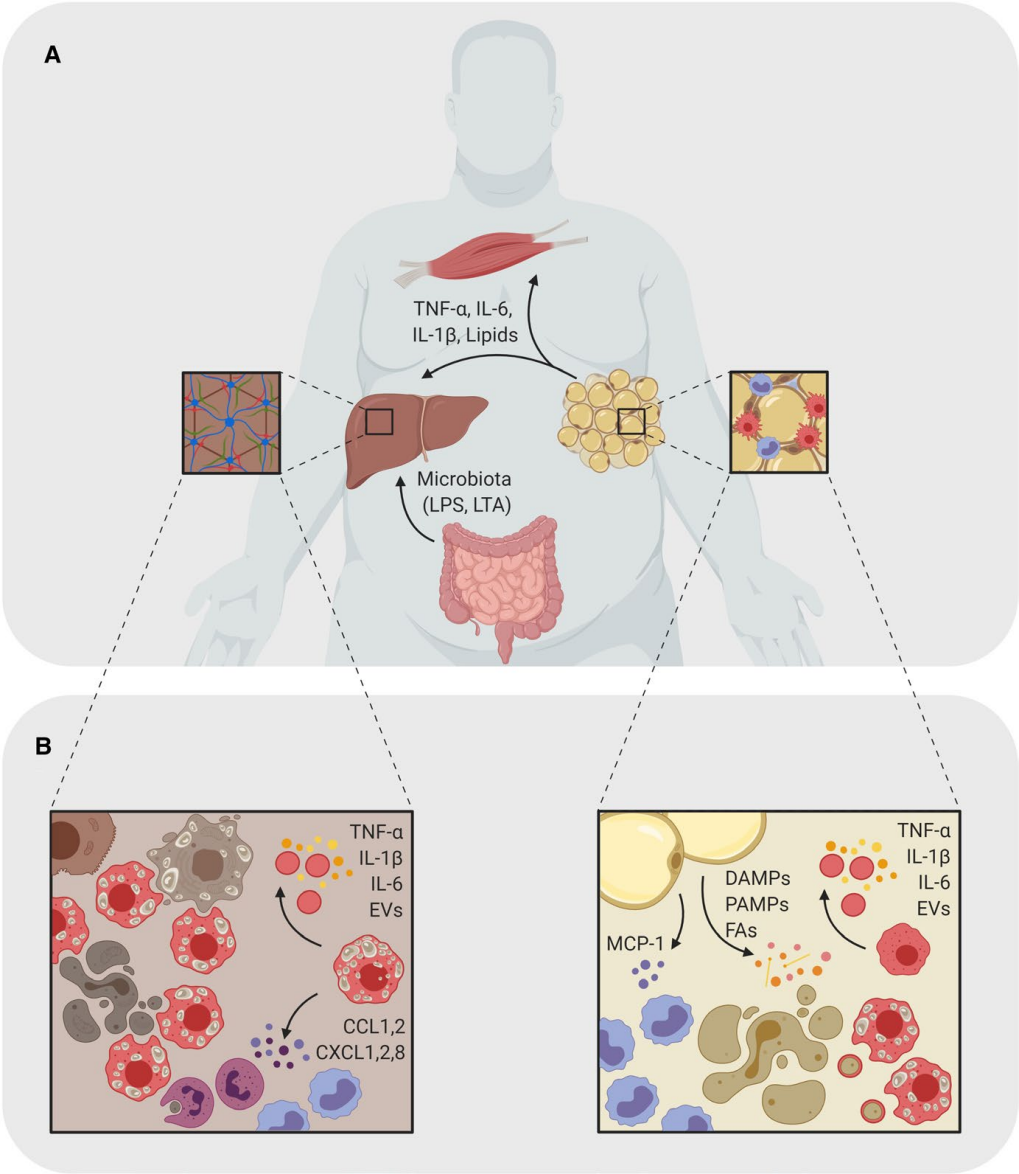
Role of macrophages in obesity‐driven NAFLD. A, Inflammatory macrophage activation (shown in red) promoting the progression of NAFLD involves a cross‐talk of AT, liver, gut and skeletal muscles. The inflammatory state in the AT leads to secretion of lipids and pro‐inflammatory cytokines into the blood stream where they travel to other metabolic organs, such as the skeletal muscles and the liver. Here, they induce an inflammatory state that can lead to metabolic diseases caused by chronic low‐grade inflammation. In obesity, the liver also receives LPS and lipoteichoic acid (LTA) from the gut, which also contribute to inflammation and tissue damage. B, During obesity, adipocytes stimulate monocyte infiltration by secreting MCP‐1. Adipocyte secretion of various DAMPs, PAMPs and FAs mediate a pro‐inflammatory polarization and differentiation of ATMs and monocytes. Within the liver, excess lipids, apoptitic bodies and AT‐derived cytokines induce an inflammatory environment with pro‐inflammatory hepatic macrophages and foam cells (shown in red). Their respond to the constant stimuli maintains the inflammatory state of the tissue by secreting pro‐inflammatory factors and neutrophil‐ and monocyte (shown in purple) attractants.

The exact pathogenesis of NAFLD is not completely understood, although a ‘multi‐hit’ hypothesis of NALFD is generally accepted, where genetic, epigenetic and environmental factors influence the disease activity, making it a very dynamic disease.^6^ Impaired function of other organs, such as disrupted gut barrier function or obese adipose tissue (AT), accelerates the progression of the disease (Figure 1A).^7^ Chronic hepatic lipid accumulation causes oxidative stress and endoplasmic reticulum stress leading to induction of inflammatory pathways, in which macrophages play an essential role (Figure 1B).^8^ However, the exact role of macrophages and the mechanisms by which metabolic stress leads to NASH with recruitment and activation of monocytes/macrophages are not fully understood. In this review, we focus on the contribution of macrophage functions to the progression of obesity‐induced NAFLD.

## 2 HEPATIC MACROPHAGES MEDIATE NAFLD‐NASH PROGRESSION

Non‐alcoholic fatty liver disease is the hepatic manifestation of metabolic syndrome and the most common chronic liver disease worldwide. It is a consequence of enhanced hepatic lipid accumulation. This excessive accumulation of hepatic fat leads to the deregulation of the immune system, activation of pro‐inflammatory signals, the breakdown of hepatic immune tolerance and development of NASH. Crucial to this transition process is hepatic macrophages.^9^, ^10^, ^11^

Hepatic macrophages are a mixture of liver‐resident Kupffer cells and monocyte‐derived macrophages. Kupffer cells are the most abundant tissue‐resident macrophage cell type in mammalian bodies, accounting for 80%‐90% of total tissue‐resident macrophages.^12^ They were first described by Karl Wilhelm von Kupffer as endothelial cells and components of liver vascular walls. They reside within the liver sinusoid where they receive portal venous blood from the gut and spleen as well as arterial blood from aorta. As such, Kupffer cells are the key sentinels at the interface of liver with other organs, such as the gut and AT.^13^ They are the first responders to liver injury by sensing danger‐associated molecular patterns (DAMPs) including fatty acids, cholesterol from AT and pathogen‐associated molecular patterns (PAMPs), such as leaky gut‐derived endotoxins.^14^

Recent data have demonstrated that Kupffer cells are predominantly from embryonic yolk sac‐derived macrophages and foetal liver monocytes with a minimal contribution from circulating blood monocytes at steady state.^15^ In mice, Kupffer cells are characterized by the specific expression of C‐type lectin domain family 4 member F (CLEC‐4F) and TIM4^+^,^16^, ^17^ while a specific marker to identify human Kupffer cells remains to be elucidated. Kupffer cells have been described as important mediators of inflammation as for example Kupffer cell‐specific deletion through administration of gadolinium chloride in vivo attenuated NASH development.^10^ However, under pathological condition, such as the progression from NAFL to NASH, there is massive recruitment of blood monocytes into the liver.^17^ As such, monocyte‐derived hepatic macrophages might be the mediators and drivers in chronic low‐grade inflammation in NAFLD. Indeed, several studies demonstrated the contribution of both Kupffer cells and recruited monocytes in the development of NASH.^18^, ^19^, ^20^ However, the underlying mechanisms and functional roles of different liver macrophage subsets are still not completely clear, given limitations due to the lack of tissue‐specific macrophage depletion models. It is still not well understood whether the microenvironment, due to excess of lipids, alters phenotype of resident Kupffer cells or provides the niche for infiltrating monocytes that differentiate into a new cell population. The recent identification of specific markers for Kupffer cells and new scientific methods (fate mapping and lineage tracing) makes it possible for a more targeted approach in the complex investigation of hepatic macrophage biology in homeostasis and disease.^21^ Overall, the question is whether the origin of recruited monocytes determines their phenotype. To understand the impact and contribution of these different monocyte and macrophage subsets in the progression from NAFL to NASH, the population and repopulation of hepatic macrophages require further investigations.

**Figure 2:**
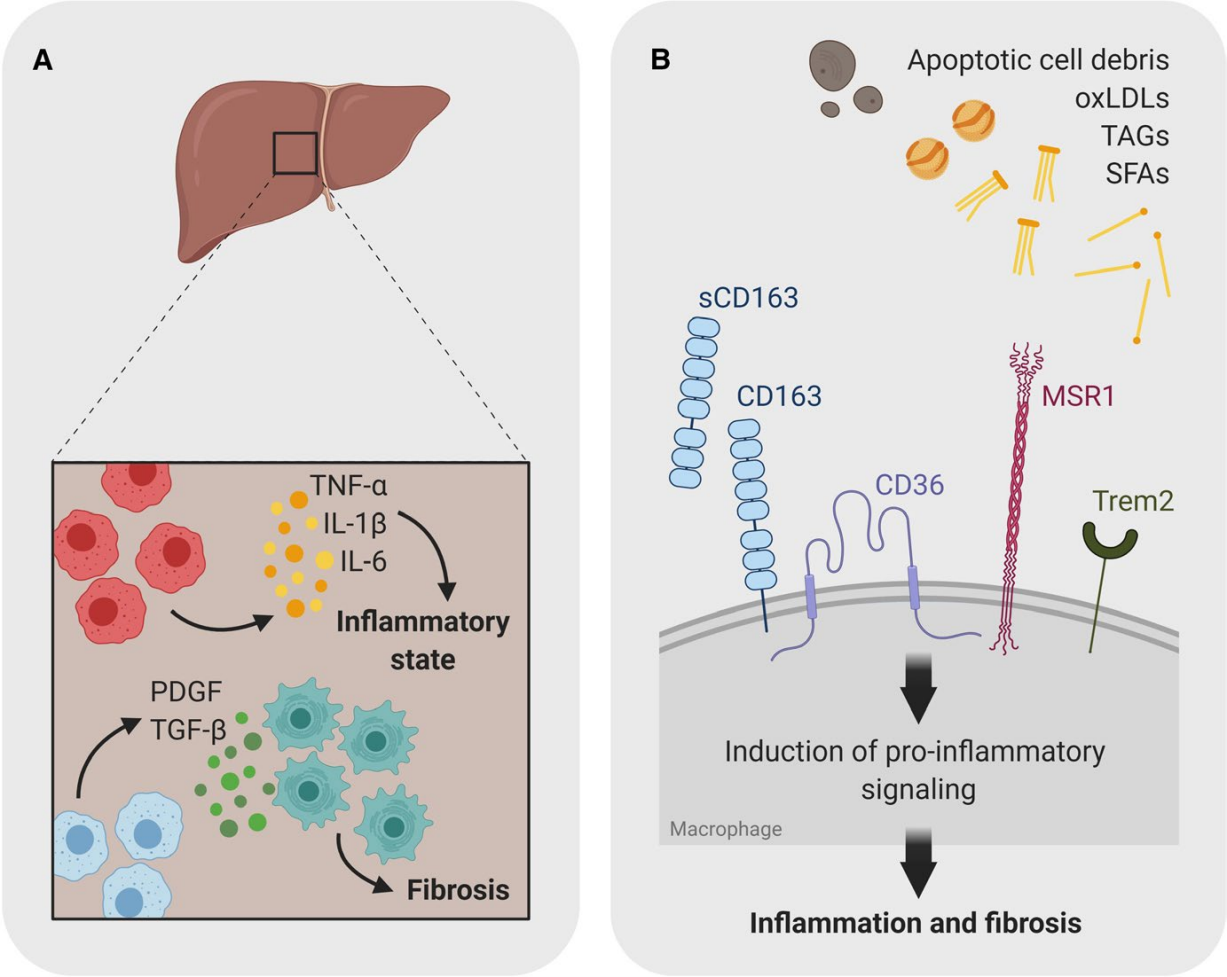
Scavenger receptors and Toll‐like receptors contribute to the inflammatory activation of macrophages. A, A variety of external cues can induce pro‐inflammatory macrophage polarization through the activation of inflammatory pathways. In obesity, these signals include a variety of lipids, cell debris, pathogen‐associated molecular patterns (PAMPs) that are recognized by scavenger receptors (SRs) and Toll‐like receptors (TLRs) on the cell surface of macrophages. Triggering these receptor contributes to the chronic low‐grade inflammation leading to tissue damage and ultimately fibrosis. B, Scavenger receptors expressed on myeloid cells are activated by apoptotic cell debris, TAGs, lipids etc Upon activation of these receptors, they induce pro‐inflammatory signalling in myeloid cells polarizing anti‐inflammatory tissue‐resident macrophages to pro‐inflammatory phenotype. Constant activation leads to a chronic low‐grade inflammation and ultimately to fibrosis

Fibrosis is a hallmark in NASH and can lead to permanent scarring of the liver, which can further develop to HCC. Hepatic stellate cells are pericytes located in the perisinusoidal space. They produce components of the extracellular matrix and are the main contributors to fibrosis.^22^ Recent studies have shown that macrophage populations in inflammatory tissue are highly heterogeneous and contain both pro‐ and anti‐inflammatory macrophages (Figure 2A).^23^ During NASH progression, hepatic stellate cells are activated by apoptotic hepatocytes, oxidative stress and pro‐inflammatory as well as anti‐inflammatory immune cells. The secretion of pro‐inflammatory cytokines and pro‐fibrotic factors, such as TGF‐β, by hepatic macrophages and infiltrating monocytes enhances the pro‐fibrotic process of the hepatic stellate cells.^24^ A potential biomarker for fibrosis is the scavenger receptor CD163. Soluble CD163 from adipose tissue macrophages (ATMs) and hepatic macrophages—which is increased in inflammatory macrophages—has been associated with fibrotic tissue.^25^ The pro‐inflammatory and pro‐fibrotic processes amplify each other and might lead to cirrhosis.

Cirrhosis is a major risk factor for the development of HCC, which is the most common type of liver cancer in adults.^26^ Macrophages contribute to the development of HCC first by inducing a chronic low‐grade inflammatory environment with oxidative stress that induces DNA damage and hepatocyte death.^27^ Infiltrating monocytes respond to the stimuli from the tumour microenvironment and differentiate into tumour‐associated macrophages (TAMs). TAMs are a heterogeneous population with a dominating anti‐inflammatory phenotype that is associated with a poor survival.^28^

## 3 ADIPOCYTE‐MACROPHAGE CROSS‐TALK

Adipose tissue is not simply an organ for lipid storage and energy homeostasis, but in fact, accumulating evidence suggests that immune cells within AT contribute to the pathophysiology of obesity‐related diseases, such as NAFLD.^17^, ^29^ It is well accepted that prolonged and excess nutrient uptake induce chronic low‐grade inflammation within the AT, contributing to the manifestation of metabolic disorder, characterized by altered production of hormones, pro‐inflammatory cytokines and adipokines (Figure 1A).^30^

Adipose tissue macrophages are believed to be one of the major players in regulating AT inflammation.^8^ Healthy AT has a limited number of resident macrophages dominated by an anti‐inflammatory phenotype. In obesity, adipocytes start to enlarge and undergo cellular hypertrophy. As a consequence of adipocyte metabolic dysfunction, they increase production of the macrophage recruitment molecule, monocyte chemoattractant protein 1 (MCP‐1 or CC‐chemokine ligand 2; CCL2), which attracts monocytes to the AT.^31^, ^32^ In addition to recruitment, MCP‐1 promotes ATM proliferation within the tissue, significantly contributing to increased ATM numbers.^33^ Further exposure of these ATMs to free fatty acids (FFAs) and triglycerides leads to their pro‐inflammatory activation and increased production of tumour necrosis factor‐alpha (TNF‐α), interleukin‐6 (IL‐6) and interleukin‐1β (IL‐1β) (Figure 1B), which has been shown to impair insulin sensitivity and glucose intolerance leading to insulin resistance, which is highly associated with other metabolic diseases as well as cancer.^34^, ^35^, ^36^, ^37^ Deletion of adipocyte MCP‐1 *in vivo* protected against obesity‐induced insulin and glucose resistance, indicating the importance of this pathway in the regulation of glucose metabolism.^31^ However, genetic deletion of the murine MCP1 receptor, CCR2, showed inconsistent results in prevention of obesity, ATM recruitment and insulin/glucose insensitivity.^38^, ^39^ These data indicate that other factors might be involved in the activation of CCR2 and monocyte recruitment.

Adipose tissue macrophages activation status determines effector functions. Toll‐like receptor 4 (TLR4) is a well‐known pathogen recognition receptor (PRR) sensing lipopolysaccharide (LPS) from Gram‐negative bacteria.^40^ In addition to LPS, TLR4 was also reported to be activated by FFAs; however, a recent in vitro study demonstrated that TLR4 is not a receptor for FFAs.^41^ Our recent work indicates that the phagocytic receptor, macrophage scavenger receptor 1 (MSR1, SR‐A), is involved in FFA uptake followed by activation of JNK signalling independent of LPS, indicating an important role of scavenger receptors in lipid‐induced inflammation.

Activated ATMs have been linked to obesity and progression of NAFLD in mouse models and humans.^42^, ^43^ In unhealthy obesity, fat mobilization from adipocytes is impaired and insulin is unable to suppress lipolysis, which is the key mediator of increased lipid flux in ectopic organs such as liver and skeletal muscle.^44^

## 4 MACROPHAGES AND LIPID SENSORS

Recently, it has been shown that different macrophage subsets display a distinct phenotype upon destabilisation of lipid homeostasis in the context of obesity. However, there is little known about the short‐ and long‐term effects on hepatic and extrahepatic complications. In recent years, single‐cell and bulk RNA sequencing have helped us enormously to better understand the pathophysiology of liver diseases. For instance, extensive single‐cell analysis of human NAFL and NASH livers defined a TREM2^+^, CD9^+^ macrophage subpopulation to be associated with fibrosis.^45^ The scavenger receptor TREM2 (triggering receptor expressed on myeloid cells 2) has been found to be a major driver of tissue‐level immune cell remodelling. TREM2 regulates genes involved in phagocytosis, lipid catabolism and energy metabolism.^45^ TREM2 has been recently linked to the lipid‐metabolizing lipid‐associated macrophages (LAMs) within AT that prevent the development of metabolic dysregulation in obesity. Additionally, role of TREM2 has been described in the development of disease‐associated microglia (DAMs) and their protective function in neurodegenerative diseases.^46^ Notably, single‐cell RNA sequencing of AT from humans and mice showed a conserved TREM2 signature similar to the gene signature of DAMs.^46^, ^47^ Common signs of adiposity, neurodegeneration and atherosclerosis are the accumulation of extracellular lipids and inflammation. Therefore, TREM2 may function as a receptor for signals upon loss of tissue homeostasis by sensing extracellular lipids during metabolic dysfunction.^46^

Another group of lipid sensors involved in lipid uptake is scavenger receptors. Scavenger receptors have been of major interest in the study of atherogenesis over the past decade and were first described in macrophages as alternative receptors to the low‐density lipoprotein (LDL) receptor in the uptake of cholesterol and lipids.^48^ Interestingly, while most of scavenger receptors are important in the clearance of oxidized low‐/high‐density lipoproteins (LDL/HDL) or apolipoproteins, the exact underlying pathways are still unknown. We have recently shown that MSR1, which is highly expressed in hepatic macrophages, plays an important immunoregulatory role in the early development of obesity‐induced NAFLD and is responsible for foam cell formation—lipid laden macrophages associated with cardiovascular diseases, such as NAFLD (Figure 2B). Moreover, our data indicate that genetic variations in MSR1 could modify protein function, macrophage phenotype and thereby NAFLD development. These data support the notion of the role of genetic factors in susceptibility to NAFLD.^41^, ^49^, ^50^ In conclusion, further characterization of scavenger receptors in the liver is thus of particular interest to better understand the pathophysiology of NAFLD and may potentially offer a powerful strategy for harnessing protective functions not only in the development and progression of NAFLD but also the metabolic syndrome in general.

## 5 CONCLUSIONS

Mounting evidence highlights the correlation of obesity with NAFLD, as the risk and severity factor for hepatic steatosis and steatohepatitis. However, there is still much to be understood regarding the mechanisms of the disease. Functional and phenotypic diversity of macrophages plays a key role in the spectrum of metabolic syndrome‐related diseases, such as NAFLD. Their function depends on their origin as well as activation, differentiation and polarization affected by both local and systemic signals in the progression of NAFLD. Moreover, there is also intertwined regulation of macrophage functions from different compartments such as circulation, bone marrow, AT, gut and liver. Mechanistically, it is of special interest to better understand the role of lipid metabolism in the regulation of macrophage function. More recently, scavenger receptors have come to focus as regulators of macrophage subsets, through which they affect inflammation and fibrosis. As such, additional studies are required to further investigate the functional role of specific macrophage subpopulations and their regulators in the form of scavenger receptors in NAFLD progression in order to target them for therapy.

## AUTHOR CONTRIBUTIONS

SKP, OGB, OG and AH wrote the manuscript and designed the figures. All authors approved the manuscript and agreed to be accountable for the content of the work.
